# Use of Chitosan–Iron Oxide Gels for the Removal of Cd^2+^ Ions from Aqueous Solutions

**DOI:** 10.3390/gels10100630

**Published:** 2024-09-30

**Authors:** Eduardo Mendizábal, Nely Ríos-Donato, Minerva Guadalupe Ventura-Muñoz, Rosaura Hernández-Montelongo, Ilse Paulina Verduzco-Navarro

**Affiliations:** 1 Department of Chemistry, CUCEI, University of Guadalajara, Blvd. Marcelino García Barragán 1421, Guadalajara 44430, Jalisco, Mexico; eduardo.mmijares@academicos.udg.mx (E.M.); nely.rios@academicos.udg.mx (N.R.-D.); 2 Department of Innovation Based on Information and Knowledge, CUCEI, University of Guadalajara, Blvd. Marcelino García Barragán 1421, Guadalajara 44430, Jalisco, Mexico; minerva.ventura@academicos.udg.mx; 3 Department of Translational Bioengineering, CUCEI, University of Guadalajara, Blvd. Marcelino García Barragán 1421, Guadalajara 44430, Jalisco, Mexico; rosaura.hernandez@academicos.udg.mx

**Keywords:** chitosan, magnetite, iron oxide, cadmium, adsorption

## Abstract

High-quality water availability is substantial for sustaining life, so its contamination presents a serious problem that has been the focus of several studies. The presence of heavy metals, such as cadmium, is frequently studied due to the increase in the contamination levels caused by fast industrial expansion. Cadmium ions were removed from aqueous solutions at pH 7.0 by chitosan–magnetite (ChM) xerogel beads and chitosan–FeO (ChF) xerogel beads in batch systems. Kinetic studies were best modeled by the Elovich model. The adsorption isotherms obtained showed an inflection point suggesting the formation of a second layer, and the BET model adjusted to liquid–solid systems was adequate for the description of the experimental data. Maximum uptake capacities of 36.97 ± 0.77 and 28.60 ± 2.09 mg Cd/g xerogel were obtained for ChM and ChF, respectively. The studied composites are considered promising adsorbent materials for removing cadmium ions from aqueous systems.

## 1. Introduction

Water is crucial for life, making its decontamination a key area of research. Heavy metals in the environment are a common issue due to their harmful effects on those who consume contaminated water. Cadmium, a heavy metal found in wastewater from industries such as metal and cement production, pigment and battery manufacturing, and petrochemical complexes, has strict regulatory limits. The Mexican Official Standard NOM-127-SSA1-2021 allows a maximum of 0.005 mg/L of cadmium in drinking water, whereas the World Health Organization reduces the limit to 0.003 mg/L [1]. In Mexico, cadmium contamination in underground waters has been reported as high as 0.019 mg/L [2] in the state of Yucatán and 0.005 mg/L in the state of Aguascalientes [3].

Various technologies and processes for removing heavy metals from water include reverse osmosis, ion exchange, chemical precipitation, chemical coagulation and flocculation, bioremediation, membrane separation and adsorption [4,5]. Adsorption stands out as an efficient and cost-effective technique, especially effective for removing low concentrations of contaminants [6,7]. Activated carbon and silica gel are commonly used for removing trace elements but are costly. Consequently, there is a growing interest in alternative sorbent materials like clays, agricultural waste, biomass, and resins. Modifications of such materials have been proposed aiming to improve their properties and uptake capacities [7]. Also, the use of hydrogel adsorbent materials for the removal of heavy metals from aqueous media has been repeatedly reported to be effective, mainly due to their three-dimensional porous network, where hydrophilic functional groups act as complexing agents for heavy metals [8].

Chitosan has been widely studied as a bio-sorbent because it is biodegradable, nontoxic, and obtained from chitin, the second most abundant natural polymer after cellulose [9,10]. Chitosan is particularly effective in removing ionic species due to its amine and hydroxyl groups, which serve as active sites for adsorption [11,12]. Chitosan is supplied in powder or flake form, but it is preferred to be used in granular forms or the form of beads for its application [13,14,15,16]. Improved functionality can be attained by combining chitosan with other materials for the obtention of composites with increased adsorption capacity [17].

The cadmium uptake capacity of chitosan was long determined to be approximately 6.4 mg/g [18], and its capacity is significantly increased when incorporating magnetic particles. Iron oxides, known for their effectiveness as adsorbents of ionic species [19], are low-cost materials that exhibit a high affinity for chitosan [20,21]. Magnetite nanoparticles of 30 and 60 nm diameters have shown cadmium uptake values of up to 228.05 mg/g and 170.86 mg/g, respectively [22]. Chitosan magnetic composites have demonstrated significantly higher cadmium uptake capacity compared to pure chitosan. Specifically, chitosan–maghemite nanoparticles achieved a maximum uptake of 15.2 mg Cd/g at pH 5 [23]. Also, a ferrofluid mediated chitosan mesoporous carbon nanohybrid reported an uptake capacity of 251.9 mg/g at pH 8 [24]. Studies on cadmium removal at pH 6 have shown uptake capacities of 127.79 mg/g, 88.5 mg/g, and 193.5 mg/g using magnetic attapulgite functionalized by chitosan and EDTA [25], magnetic kaolinite immobilized chitosan beads [26], and phenyl-aminophosphonate chitosan–magnetic nanocomposite [27], respectively. A more recent study at pH 5 using a magnetite–chitosan composite reported an uptake capacity of 18.67 mg/g [28], while a 200 mg/g cadmium uptake capacity was achieved with thiazole derivative-grafted chitosan–magnetite nanoparticles at the same pH [29]. At lower pH values, such as pH 4, uptake capacities of up to 300 mg/g have been reported using hydrazide micro magnetite chitosan [30]. While optimum cadmium adsorption has been reported to occur at approximately pH 5 and 6 using chitosan composites, this study focused on removing cadmium ions from aqueous media at pH 7, using xerogel beads of chitosan–magnetite (ChM) and chitosan–ferrous oxide (ChF) in batch systems. This pH was chosen as it is representative of most natural waters, which typically have a pH range of 6–9 [31]. Chitosan has been reported to have a maximum Cd^2+^ uptake capacity value of 6.4 mg/g at pH 8 [18] while for magnetite a value of 228 mg/g was reported at pH 6 [22]. However, a study reported recently indicates that the optimum pH for cadmium removal with magnetite is pH 7 [32]. Therefore, the present work was performed at pH 7 to study cadmium removal with ChM and ChF composite materials considering that magnetite, despite being a minor component in the composite compared to chitosan, it has a much higher cadmium adsorption capacity.

## 2. Results and Discussion

### 2.1. Xerogel Beads Characterization

#### 2.1.1. Size and Composition of the Xerogel Beads

The ash contents in ChM, ChF, and chitosan xerogel beads were 10.3 ± 0.4%, 10.2 ± 0.3%, and 1.4 ± 0.1%, respectively. With this information, the amount of magnetite in ChM was calculated to be 8.9 ± 0.4%, and that of FeO in ChF was 8.8 ± 0.3%.

#### 2.1.2. Average Diameter of Gel Beads

ChM xerogel beads had an average diameter of 1.14 ± 0.05 mm, and ChF beads 1.17 ± 0.04 mm. After swelling, the average diameters of the ChM beads were 1.23 ± 0.04 mm and that of the ChF beads 1.28 ± 0.07 mm.

#### 2.1.3. Scanning Electron Microscopy (SEM)

**Figure 1:**
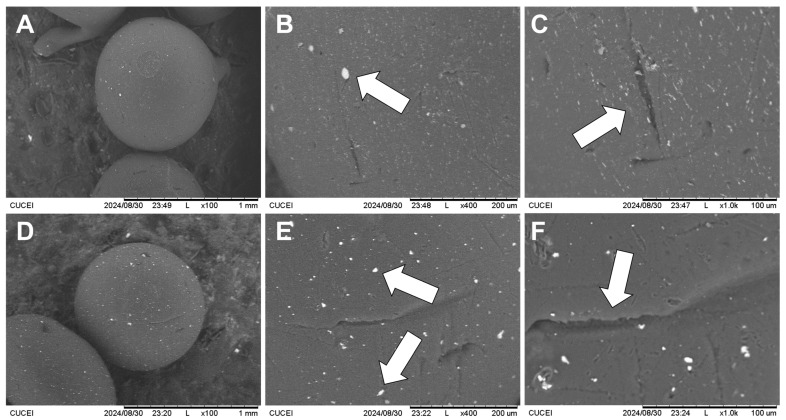
SEM images with: 100× magnification of (**A**) ChM and (**D**) ChF, 400× magnification of (**B**) ChM and (**E**) ChF, and 1000× magnification of (**C**) ChM and (**F**) ChF.

Micrographies obtained from SEM analyses of ChM and ChF xerogel beads are shown in Figure 1. Xerogel beads present a spherical shape, as can be observed in Figure 1A,D for ChM and ChF, respectively. In Figure 1B,E, clusters of magnetite particles (in ChM) and FeO (in ChF) are unevenly dispersed within the chitosan matrix, as indicated by arrows in the images. Also, an irregular surface is observed in both composite materials, where cracks are signaled in Figure 1C,F.

#### 2.1.4. Potentiometric Titration

**Figure 2:**
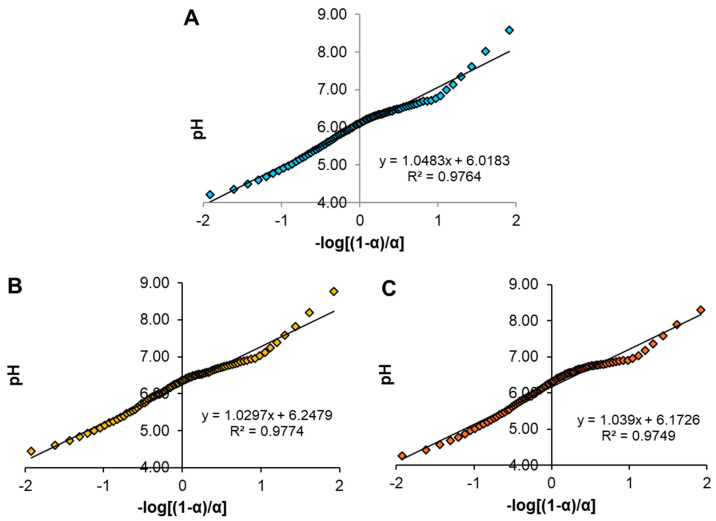
Potentiometric titration for the determination of the zero charge point of (**A**) chitosan, (**B**) ChM, and (**C**) ChF.

The graphs obtained from the titrations of chitosan, ChM, and ChF are shown in Figure 2, where it was determined graphically that the pKa of chitosan was 6.01 (Figure 2A). Figure 2B,C present the titration data of the ChM and ChF, where it is observed that their pKa value shifted to 6.25 and 6.17, respectively. Using the Henderson-Hasselbalch equation (Equation (1)) it is determined that at a pH of 7.0, the percentage of protonated amine groups of ChM and ChF would be 15.10% and 12.88%, respectively.

#### 2.1.5. Point of Zero Charge Determination

**Figure 3:**
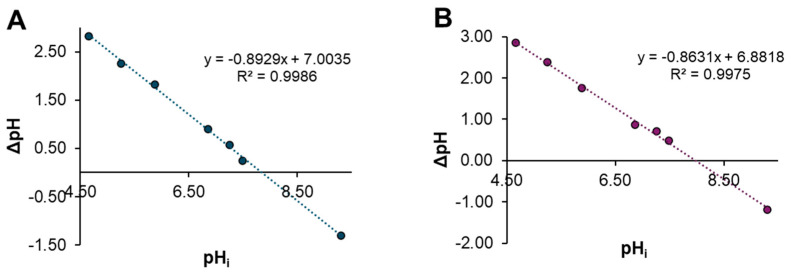
Plots of the change in pH (ΔpH) as a function of pH_i_ using the salt addition method: (**A**) ChM and (**B**) ChF.

The composite materials’ zero-point charge (PZC) was obtained graphically. Figure 3 shows the change in the pH value of the aqueous media after being in contact with ChM and ChF, where the pH_PZC_ for ChM was determined to be 7.84 for and 7.97 for ChF, which indicates that the gel beads had a positive net charge during the sorption process at the studied pH. It has been previously reported that chitosan has a pH_PZC_ of 8.0 [33], so it is expected that at pH < pH_PZC_, the protonated groups would be chitosan’s amine group; however, as stated previously from pKa values, only approximately 12.88 to 15.10% of the amine groups are protonated, so the rest of the amine groups remain unprotonated and are able to interact with the Cd^2+^ ions. Complementarily, it has been reported that the pH_PZC_ of magnetite and FeO are 4.0 [34] and 4.3 [35], respectively, which means that at pH 7, pH > pH_PZC_, ion oxides are mainly found in the form of –FeO^−^ [34], and can interact with Cd^2+^ ions.

#### 2.1.6. X-ray Diffraction Crystallography

**Figure 4:**
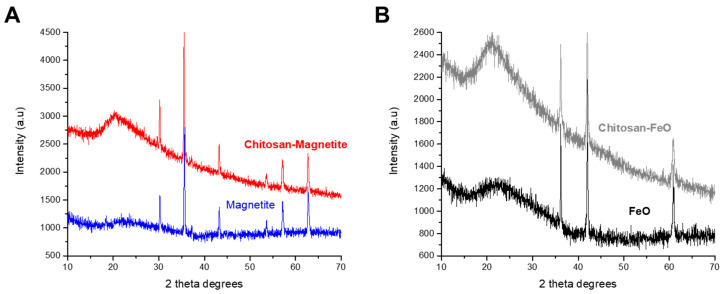
X-ray diffraction pattern of (**A**) magnetite and ChM and (**B**) FeO and ChF.

Figure 4A shows the diffraction patterns of magnetite and ChM, where the characteristic peaks of magnetite at 30, 35, 43, 53, 57 and 62 2θ degrees, corresponding to the diffraction planes (220), (311), (400), (422), (511) and (440) are also present in the ChM confirming the presence of magnetite oxides in the ChM. In Figure 4B, it can be observed the diffraction patterns of FeO, where the intensity signals at 36, 41 and 60 2θ degrees are also present in the ChF, confirming its presence in the ChF [36].

#### 2.1.7. FTIR Characterization

**Figure 5:**
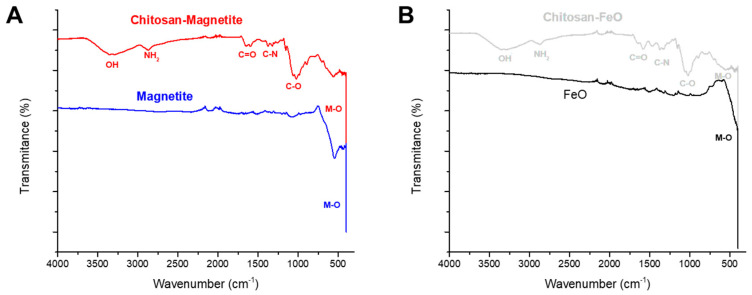
FTIR spectra of (**A**) magnetite and ChM, (**B**) FeO and ChF.

The FTIR spectra for ChM and ChF samples were analyzed (Figure 5). In the ChM spectrum (Figure 5A), high-intensity bands are observed at 3348 and 2870 cm^−1^, corresponding to the hydroxyl groups and primary amines in chitosan, respectively. The band at 1624 cm^−1^ is due to the asymmetric stretching vibrations of the carbonyl from the amide, and the band at approximately 1300–1380 cm^−1^ is attributed to C—N bending vibrations. The pyranose ring’s C—O stretching vibrations are present at 1023 cm^−1^. The intense peak at 500–450 cm^−1^ indicates the presence of the magnetite [37,38]. The ChF spectra (Figure 5B) closely resemble that of the ChM, and the presence of the FeO is confirmed by the peak between 500 and 400 cm^−1^ [37].

### 2.2. Adsorption Kinetics

**Figure 6:**
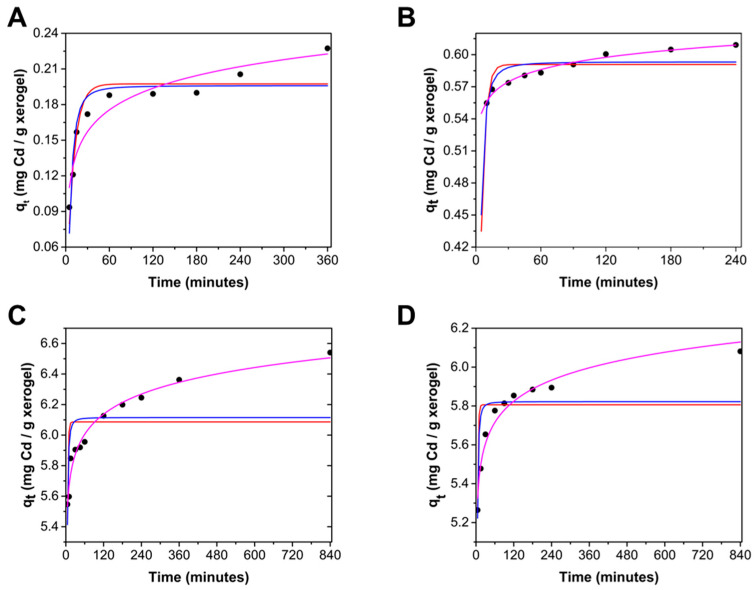
Experimental kinetic data of Cd sorption (•) using different initial cadmium concentrations: (**A**) 10 mg/L ChM, (**B**) 10 mg/L ChF, (**C**)100 mg/L ChM, and (**D**) 100 mg/L ChF. Predicted values by Elovich (**—**), pseudo-first-order (**—**) and pseudo-second-order (**—**) models.

Figure 6 illustrates the adsorption kinetics of Cd using ChM and ChF hydrogels. Initially, there is rapid adsorption, then slows down significantly, though adsorption continues throughout the experiment. After 30 min, with an initial Cd concentration of 10 mg/L, 18% of Cd was adsorbed by the ChM and 53% by ChF, and with an initial concentration of 100 mg/L, 58% of Cd was adsorbed by both adsorbents.

Experimental data of cadmium adsorption kinetics by ChM and ChF beads at 25 °C and pH 7.0 were fitted using three models (Table 1): pseudo-first order (Equation (3)), pseudo-second order (Equation (4)) and Elovich (Equation (5)). Nonlinear regression analysis revealed that the pseudo-first and pseudo-second-order models did not fit the data due to low determination coefficients (R^2^) and higher mean average error (MAE) values. The Elovich model had lower MAE and higher R^2^ values, indicating it is the best model for predicting Cd uptake by the hydrogel beads. This model suggests that the surface of the adsorbent is energetically heterogeneous [39], which can be attributed to the presence of diverse functional groups (amine from chitosan and hydroxyl and oxygens from the iron oxides) able to interact with cadmium cations. Figure 6 confirms that the Elovich model best fits the experimental data. The Elovich model predicts that adsorption does not reach equilibrium, although the increase in adsorption is minimal at long times, which is also evident in Figure 5.

### 2.3. Adsorption at Equilibrium

**Figure 7:**
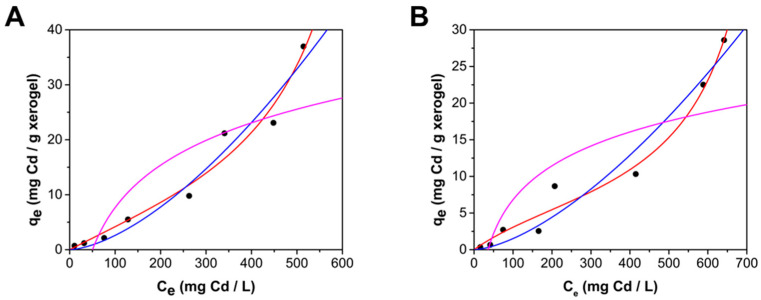
Cadmium adsorption onto (**A**) ChM and (**B**) ChF at pH 7.0 and 25 °C; experimental (-) and predictions by BET (**—**), Freundlich (**—**) and Temkin (**—**) models.

Figure 7 shows the adsorption isotherm of cadmium on ChM and ChF, indicating an S-type isotherm rather than the L-type observed by Hu et al. [28] using a magnetite–chitosan composite. This variation is attributed to the different cadmium concentration ranges studied. This work studied concentrations up to 514 mg Cd/L, whereas the study reported by Hue et al. only went up to 180 mg Cd/L. Figure 6 shows that a second adsorption layer forms at concentrations above 250 mg Cd/L, suggesting that the different isotherm behavior reported by Hu et al. [28] might be due to the inability to form a second layer at the lower concentration range examined.

The obtention of S-type isotherms could be a result of two opposite mechanisms [40], meaning a more complex adsorption process, which has been reported previously for arsenate adsorption onto ChM [41]. Also, a cooperative adsorption phenomenon could occur in this type of isotherms, which would be consistent with multilayer adsorption, where the initial layer becomes a surface for further adsorption [42].

Because the adsorption isotherms are S-type, the adsorption equilibrium data were fitted by nonlinear regression analysis to the Freundlich, the Temkin, and the BET models. The Freundlich model (Equation (6)) assumes that the adsorption occurs on a heterogeneous surface, unlike the Langmuir model, which assumes that all sites are energetically equivalent and the surface is uniform [39,43]; the Temkin model (Equation (8)) assumes that the adsorption is characterized by a uniform distribution of the binding energies; the BET model is noted for its success in describing multilayer adsorption, but the classical BET equation, initially developed for gas phase adsorption, can lead to errors when applied to liquid phase adsorption. Therefore, a BET equation adapted for liquid phase adsorption [44] was used in this study. Table 2 presents the estimated parameters for the three models. The highest correlation coefficient values and the lowest MAE values indicate that the adapted BET model for liquid phase adsorption (Equation (7)) best describes the experimental data.

The BET model predicts q_m1_ values of 13.97 and 7.02 mg Cd/g xerogel for ChM and ChF, respectively, values corresponding to the formation of an unimolecular layer of Cd, which shows as an inflection in the adsorption isotherms (Figure 7). Table 3 shows that the maximum adsorption values obtained (q_e_) were 36.97 mg Cd/g xerogel for ChM and 28.60 mg Cd/g xerogel for ChF, respectively, and that higher cadmium uptake has been obtained with ChM compared with ChF. Table 3 also shows that the incorporation of iron oxide particles into chitosan increases the adsorption capacity of the hydrogels and that the present work presents values similar to those reported for chitosan–Fe composites.

**Figure 8:**
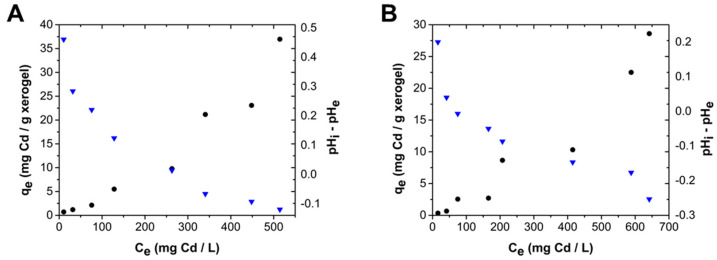
Uptake capacity (•) and change in pH (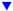) behavior as a function of cadmium concentration at equilibrium conditions at an initial solution pH of 7.0 and 25 °C. (**A**) ChM, (**B**) ChF.

The formation of a second layer and cooperative adsorption can be supported when observing the change in pH at the different cadmium concentrations at equilibrium. Figure 8 shows a decrease in pH at equilibrium conditions with increasing C_e_, which suggests that additional protons are transferred to the aqueous media when the material’s surface seems to reach saturation; this riddance of protons on amine groups leads to the creation of more available active sites to interact with cadmium cations [21], which explains the new increase in uptake capacity, observed in the inflection of the adsorption isotherm.

**Figure 9:**
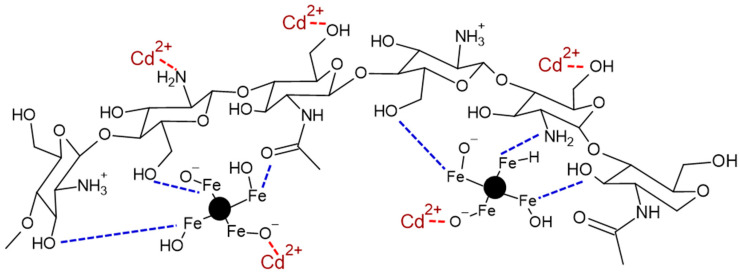
Scheme for the adsorption of Cd^2+^ onto either ChM or ChF. Interactions between chitosan and iron oxides (magnetite or FeO) in the composites are represented by (---) and the interaction between the composites and Cd^2+^ is represented by (---).

The interactions between chitosan and iron oxides (in the composites), and the interactions between the composites with cadmium ions are presented in Figure 9. It has been reported that chitosan interacts with iron atoms mainly through its amine groups [41]; however, its other groups, such as hydroxyl and carbonyl groups, are also capable of interacting with the iron atoms [48]. Cadmium ions can interact with functional groups that are negatively charged or those containing unpaired electron pairs. Because the pH_PZC_ of ChM and ChF (7.84 and 7.97, respectively) is higher than the pH interval studied during the sorption process (7.0 to 7.5), the net charge of both composites is positive. However, only approximately 12.88 to 15.10% of the amine groups are protonated, allowing the rest to adsorb cadmium ions, since cadmium ions bind with unpaired electrons in the amine and hydroxyl groups of chitosan [41,48]. Additionally, the pH_PZC_ values of magnetite and FeO are 4.0 [34] and 4.3 [35], respectively, indicating the presence of negatively charged groups –FeO^−^, which can interact with the cadmium ions. When negatively charged –FeO^−^ groups are present, cadmium ions are adsorbed through electrostatic adsorption and ion exchange by the oxygen atoms from iron oxides such as magnetite or ferrous oxide [34].

The adsorption mechanism of metals on chitosan and iron oxides is due to electrostatic attractions, which are relatively weak. It has been reported that for the removal of Cd^2+^ using chitosan at temperatures between 25 and 50 °C the Gibbs free energy ranges from 2.1 to 2.7 kJ/mol [28], which means that changing the temperature within a practical range does not significantly modify the amount of cadmium adsorbed.

### 2.4. Effect of Ionic Strength on Cadmium Uptake

**Figure 10:**
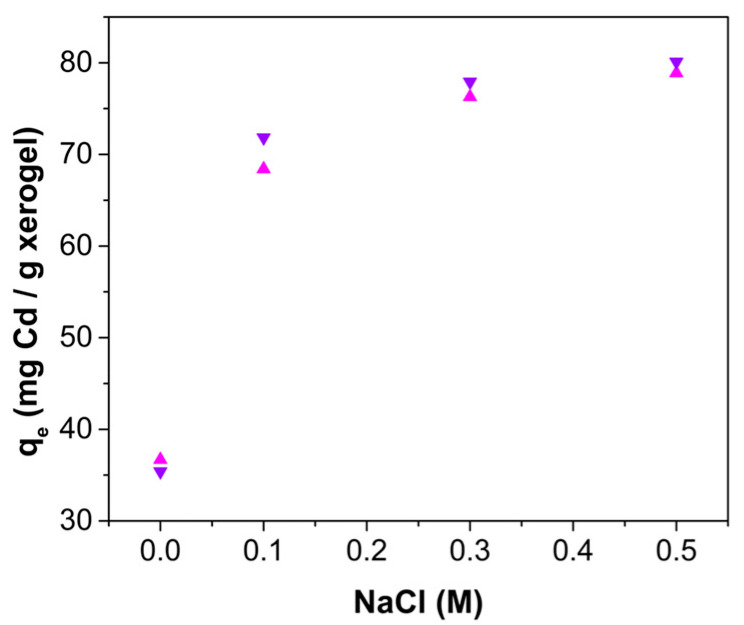
Cadmium uptake capacity of ChM (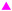) and ChF (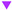) as a function of salinity, expressed as NaCl molarity.

The effect of ionic strength on the adsorption of cadmium was studied by comparing the cadmium uptake capacity obtained using a cadmium aqueous solution with no other salts added, with cadmium aqueous solutions with NaCl added (0.1, 0.3 and 0.5 M). Figure 10 shows the uptake capacity as a function of NaCl concentration, and it is observed that the adsorption of cadmium increases with increasing ionic strength. It is known that an increase in ionic strength results in a double layer with a decreased thickness, so the repulsion between charged adsorbate molecules is diminished and it results in an increased uptake capacity [49,50].

## 3. Conclusions

This study investigated the adsorption of cadmium ions at an initial pH of 7.0 and 25 °C using two types of xerogel beads: ChM with 8.9% magnetite and an average diameter of 1.14 mm, and ChF with 8.0% FeO and an average diameter of 1.17 mm. FTIR and X-ray diffraction confirmed the presence of magnetite and FeO.

The adsorption isotherms for cadmium on both ChM and ChF beads were of the S type, indicating the formation of a second adsorbate layer. The complexity of the adsorption process was best described by the multilayer BET model adjusted for liquid systems. The maximum uptake capacities were 36.97 mg Cd/g for ChM and 28.60 mg Cd/g for ChF. The adsorption of Cd increases with increasing ionic strength.

The adsorption of Cd on ChM and ChF hydrogels is initially very rapid, adsorbing over 55% of the Cd, then slows down but continues throughout the experiment. The Elovich model best describes the kinetics of Cd adsorption.

The high cadmium adsorption capacity combined with a rapid adsorption process leads to the conclusion that ChM and ChF are promising and practical materials for cadmium removal.

## 4. Materials and Methods

### 4.1. Materials

Food-grade chitosan (90% degree of deacetylation) was obtained from América Alimentos (Zapopan, México). Magnetite, in the form of nanopowder with a particle size of 50–100 nm from Sigma-Aldrich (Shanghai, China), was used as received. Iron(II) oxide was purchased from Sigma-Aldrich (Prague, Czech Republic) and was ground using an agate mortar before use. Acetic acid was obtained from Fermont (Monterrey, México). Sodium hydroxide, hydrochloric acid and cadmium chloride as CdCl_2_ · 2.5H_2_O were purchased from Golden Bell (Zapopan, México).

### 4.2. Preparation of ChM and ChF Xerogel Beads

**Figure 11:**
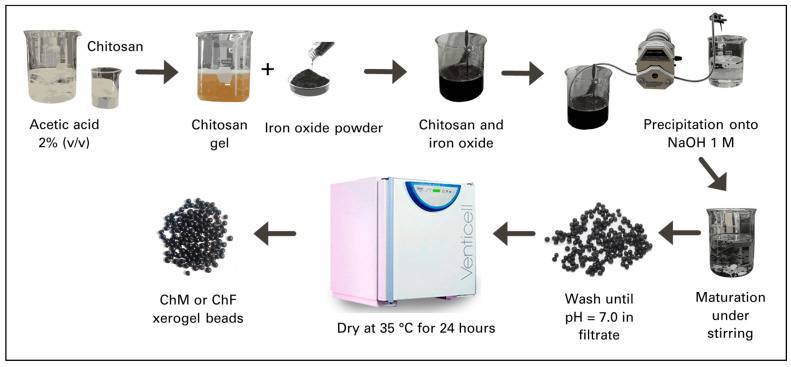
Scheme for the experimental procedure used for the obtention of ChM and ChF xerogel beads.

To ensure uniformity and repeatability of the materials used in the adsorption tests, a large batch of ChF and ChM hydrogels was prepared. After preparation, these hydrogels were kept in an aqueous solution. For the adsorption tests, each time that the hydrogels were used, the amount necessary of the hydrogels was carefully removed from the solution, dried for characterization, and used in the adsorption and equilibrium tests. The hydrogels remained in the aqueous environment for almost three years without any signs of degradation during this period. Figure 11 summarized the experimental procedure used for the obtention of ChM and ChF xerogel beads. A solution was prepared by adding 4.5 g of chitosan powder to 100 mL of 2% acetic acid. Subsequently, 0.45 g of magnetite or FeO powder was added and mixed into the chitosan solution with a hand-held processor until a uniform dispersion was obtained. The ChM and ChF hydrogel beads were formed by adding each dispersion dropwise into 1 M sodium hydroxide solution using a Masterflex 075557 (Gelsenkirchen, Germany) peristaltic pump and a Masterflex L/S 14 silicone hose. ChM and ChF beads were matured in the NaOH solution for 24 h, then rinsed with bi-distilled water until the filtrate’s pH was neutral (7.0). Hydrogel beads were then filtered using a plastic strained, and then were placed onto filter paper and dried at 35 °C for 24 h in a MMM Vencticell (Jurong, Singapore).

### 4.3. Xerogel Beads Characterization

#### 4.3.1. Xerogel Beads Composition

Xerogel beads were ground into powder, and 0.5 g samples were burnt at 1000 °C for 4 h in a FE-340 Muffle (Zapopan, Mexico) to obtain the ashes. Similarly, 0.5 g of chitosan was also burnt at the same temperature to determine its ash content These procedures were performed in duplicate.

#### 4.3.2. Average Diameter of Gel Beads

The diameter of ChM and ChF xerogel beads was measured using a digital electronic calibrator on a sample of 30 beads. For the determination of the average diameter of the hydrogel beads, xerogel beads were allowed to swell for 48 h in NaCl 0.01 M aqueous solutions at pH of 7.5.

#### 4.3.3. Scanning Electron Microscopy

The ChM and ChF xerogel beads were observed on a Hitachi TM 1000 scanning electron microscope (Tokyo, Japan). Analyses were obtained using operation conditions of 15.0 kV of acceleration voltage and an emission current of 48 mA.

#### 4.3.4. Potentiometric Titration

The chitosan, ChM, and ChF pKa values were determined through potentiometric titration, following the method described by Ríos-Donato et al. [51]. A 0.100 g sample of ChM or ChF was suspended in a 0.1 M HCl solution and titrated with 0.1 M NaOH. pH measurements were obtained by using an Ohaus Starter 2100 potentiometer (Parsippany, NJ, USA). Using Henderson-Hasselbalch equation (Equation (1)) [51], the pKa of the materials was determined, where α is the dissociation coefficient (α = (V − V_1_)/(V_2_ − V_1_)).

(1) $$pH={pK}_{a}+\log\left(\frac{1-\alpha }{\alpha }\right)$$

#### 4.3.5. Point of Zero Charge Determination

The zero point charge of ChM and ChF was obtained using the method reported by Udoetok et al. [52]. For this purpose, a 0.01 M NaCl stock solution was prepared, and 50 mL portions were transferred to seven beakers. The pH of the solutions in the beakers was adjusted to a range of 4.5–9.0 using 0.1 M NaOH and HCl solutions. An amount of 0.10 g of xerogel and 10 mL of 0.01 M NaCl solution at the required pH were placed in a 15 mL centrifuge tube. The tubes were then continuously shaken at 25.0 ± 0.5 °C and 100 RPM in a thermoshaker for 48 h. The liquid phase was separated by decantation, and pH was measured using an Ohaus Starter 2100 potentiometer (Parsippany, NJ, USA). The pH at the point of zero charge (PZC) for each material was determined where ΔpH equals zero in a graph of the change in pH (ΔpH) against pH.

#### 4.3.6. X-ray Diffraction Crystallography

The crystalline structure of the composites was studied by X-ray diffraction crystallography using a Panalytical model Empyream diffractometer (Malvern, England) with a copper anode radiation (kα) at a wavelength of 0.154 nm within the range of 10–70 2θ degrees. The diffractograms were collected at a pace of 0.02 degrees measured at 20 s per pace.

#### 4.3.7. FTIR Analyses

ChM and ChF were analyzed using Fourier Transform Infrared Spectroscopy (FTIR) on a Perkin Elmer spectrometer (Port Melbourne, Australia). An amount of 5 mg of samples were mixed with 100 mg of KBr, ground, and placed in a sample cup. The samples were scanned in the range of 4000–450 cm⁻^1^.

### 4.4. Adsorption Kinetics

Aqueous solutions with cadmium concentrations of 10 and 100 mg Cd^2+^/L were prepared using CdCl_2_·2.5H_2_O and bi-distilled water, with the pH adjusted to 7.00 ± 0.05 using 0.1 M NaOH. Xerogel beads (0.10 g) were added to 10 mL of the cadmium solution in centrifuge tubes, which were then placed in an MCR Thermoshaker (Port Melbourne, Australia) at 25.0 ± 0.5 °C with continuous agitation at 100 RPM. At different contact times, going from 5 min to 14 h, the solutions were separated from the beads by decantation. This procedure was performed in duplicate with each material (ChM and ChF) and both initial cadmium concentrations (10 and 100 mg/L). The amount of Cd^2+^ remaining in the solution (q_t_) was determined using a Varian SpectraAA 220 flame atomic absorption equipment (Markham, ON, Canada) at a wavelength of 228.8 nm. Equation (2) calculates the adsorption capacity at time t, where Ci and Ct are the initial and cadmium concentrations at time t, respectively. V is the volume of the solution, and m is the xerogel mass.

(2) $$q_{t}=\frac{V\left(C_{i}-C_{t}\right)}{m}d$$

Mathematical models were used to fit the experimental data using nonlinear regression analysis at a 95% significance level with OriginPro 2016 software, employing the Marquardt algorithm. The models tested were the pseudo-first-order, pseudo-second-order, and Elovich models.

(3) $$q_{t}=q_{e}\left(1-e^{-k_{1}t}\right)$$

(4) $$q_{t}=\frac{q_{e}k_{2}t}{k_{2}t+\frac{1}{t}}$$

(5) $$q_{t}=\frac{1}{\beta }\ln\left(\alpha \beta \right)+\frac{1}{\beta }\ln\left(t\right)$$

where q_t_ is the adsorption capacity at time t, k_1_ and k_2_ are the kinetic constants for the pseudo-first-order and pseudo-second-order models, respectively. α is the desorption constant and β is the initial adsorption rate [42,53,54].

### 4.5. Adsorption at Equilibrium

Cadmium aqueous solutions with concentrations, ranging from 20 to 800 mg Cd^2+^/L, were prepared from a 1000 mg Cd^2+^/L using a stock solution and bi-distilled water, both with the pH adjusted to 7.00 ± 0.05 using 0.1 M NaOH and 0.1 M HCl solutions.

ChM or ChF xerogel beads (0.10 g) were added to 10 mL of the cadmium solution in centrifuge tubes, which were then placed in an MCR Thermoshaker (Accesolab, Port Melbourne, Australia) at 25.0 ± 0.5 °C with continuous agitation at 100 RPM. After 24 h of continuous agitation, the solutions were separated from the beads by decantation. All treatments were performed in triplicate. The amount of Cd^2+^ remaining in the solution was determined using the Varian SpectraAA 220 flame (Markham, ON, Canada) atomic absorption equipment at a wavelength of 228.8 nm. The pH of the samples was measured using an Ohaus Starter 2100 pH meter.

The experimental isotherm data were fitted to the Freundlich model (Equation (6)), the modified BET model for a liquid–solid system (Equation (7)), and the Temkin model (Equation (8)) through nonlinear regression analysis using the Marquardt algorithm and the OriginPro 2016 software.

(6) qe=KFCe1n

(7) $$q_{e}=q_{m1}\frac{K_{L}C_{e}}{\left(1-K_{U}C_{e}\right)\left(1-K_{U}C_{e}+K_{L}C_{e}\right)}$$

(8) $$q_{e}=\frac{RT}{b_{T}}\ln\left(A_{T}C_{e}\right)$$

where C_e_ represents the equilibrium cadmium concentration in the liquid phase, q_m1_ represents the amount adsorbed by the adsorbent when a complete unimolecular layer covers it, and q_e_ is the cadmium adsorption capacity at equilibrium. K_F_ is the Freundlich model constant, 1/n indicates the heterogeneity factor, K_L_ is the adsorption equilibrium constant for the first layer in the BET model, and K_U_ is the adsorption equilibrium constant for the upper layers in the BET isotherm. A_T_ is the Temkin isotherm equilibrium binding constant, b_T_ is the isotherm constant, R is the universal gas constant, and T is the temperature [43,44,55,56].

### 4.6. Effect of Ionic Strength

The influence of the ionic strength on the adsorption process was studied by using a cadmium solution (900 mg Cd^2+^/L) with the pH adjusted to 7.00 ± 0.05 using 0.1 M NaOH and 0.1 M HCl solutions. Solutions with varying NaCl concentrations (0, 0.1, 0.3, and 0.5 M) were used. ChM or ChF xerogel beads (0.10 g) were added to 10 mL of the cadmium solutions in centrifuge tubes, which were then placed in an MCR Thermoshaker (Accesolab, Port Melbourne, Australia) at 25.0 ± 0.5 °C with continuous agitation at 100 RPM. After 24 h of continuous agitation, the solutions were separated from the beads by decantation. All treatments were performed in triplicate. The amount of Cd^2+^ remaining in the solution was determined using the Varian SpectraAA 220 flame (Markham, ON, Canada) atomic absorption equipment at a wavelength of 228.8 nm.

## Figures and Tables

**Table 1:** Cadmium adsorption kinetic data by ChM and ChF hydrogel beads. The experiments were conducted at 25 °C and pH of 7.0, using two cadmium concentrations (10 and 100 mg Cd/L). The uptake capacity is expressed as milligrams of Cd per gram of xerogel.

	ChM		ChF	
Kinetic Models	C_0_ = 10 mg/L	C_0_ = 100 mg/L	C_0_ = 10 mg/L	C_0_ = 100 mg/L
Pseudo-first model				
q_e_ (mg Cd/g)	0.1974	5.806	0.5908	6.086
k_1_ (1/min)	0.1034	0.4713	0.2664	0.4520
R^2^	0.8804	0.5433	0.5201	0.3192
MAE	0.0112	0.1123	0.0105	0.2065
Pseudo-second order				
q_e_ (mg Cd/g)	0.1959	0.0575	0.5932	6.115
k_2_ (g/mg·min)	0.0231	0.3484	0.1259	0.3087
R^2^	0.8437	0.6341	0.6553	0.4515
MAE	0.0132	0.1041	0.0090	0.1881
Elovich				
α	3.46 × 10^−1^	1.80 × 10^13^	6.91 × 10^11^	1.77 × 10^11^
β	38.013	6.381	60.500	5.271
R^2^	0.9066	0.9669	0.9824	0.9760
MAE	0.0110	0.0382	0.0018	0.0362

**Table 2:** Freundlich, BET and Temkin model constants, their determination coefficients and MAE in Cd adsorption on ChM and ChF hydrogel beads at 25 °C and initial pH = 7.0. Uptake capacities are expressed in Cd milligrams per gram of xerogel.

Isotherm Models	ChM	ChF
Experimental q_max_ (mg Cd/g)	36.97 ± 0.77	28.60 ± 2.09
BET model		
q_m1_ (mg Cd/g)	13.97	7.02
K_L_ (L/mg)	3.05 × 10^−3^	5.48 × 10^−3^
K_U_ (L/mg)	1.32 × 10^−3^	1.20 × 10^−3^
R^2^	0.9645	0.9715
MAE	1.533	1.038
Freundlich model		
K_F_ (L/mg)	1.85 × 10^−3^	1.25 × 10^−3^
n	0.635	0.6483
R^2^	0.9561	0.9484
MAE	2.006	1.636
Temkin model		
α	0.0199	0.0278
β	222.96	371.80
R^2^	0.7452	0.6397
MAE	6.605	4.764

**Table 3:** Cadmium uptake capacity values reported for removing Cd(II) using different chitosan composites.

Adsorbent	q_max_ (mg Cd/g)	pH	Reference
Chitosan	6.8	8	[18]
Magnetite	228	6	[22]
Chitosan–maghemite nanoparticles	15.2	5	[23]
Magnetite–chitosan	18.7	5	[28]
Magnetic chitosan cross-linked with κ-carrageenan	84.3	7.6	[45]
Magnetic kaolinite-immobilized chitosan beads	88.5	6	[26]
Chitosan iron (III) oxide nanocomposite	86.0	7	[46]
Methyl-aminophosphonate chitosan–magnetic nanocomposite	118.1	6	[27]
Chitosan–stabilized nano-zero-valent iron	124.74	6	[47]
Magnetic attapulgite functionalized by chitosan and EDTA	127.79	6	[25]
ChM	36.97	7	This work
ChF	28.60	7	This work

## Data Availability

The original contributions presented in the study are included in the article, further inquiries can be directed to the corresponding author.
